# Integrating germline and tumor sequencing to improve hereditary cancer diagnosis and care

**DOI:** 10.1038/s41431-026-02046-5

**Published:** 2026-02-23

**Authors:** Richarda M. de Voer, Laura Valle

**Affiliations:** 1 Department of Human Genetics, Radboud University Medical Center, Nijmegen, the Netherlands; 2 Radboud Institute for Medical Innovation, Nijmegen, the Netherlands; 3 European Reference Network on Genetic Tumor Risk Syndromes (ERN GENTURIS), Nijmegen, the Netherlands; 4 Hereditary Cancer Program, Catalan Institute of Oncology; Oncobell Program, IDIBELL, Hospitalet de Llobregat, Barcelona, Spain; 5 Centro de Investigación Biomédica en Red de Cáncer (CIBERONC), Madrid, Spain

**Keywords:** Cancer genetics, Cancer genetics, Biomarkers

## Abstract

A subset of cancers arises due to inherited germline pathogenic variants in specific genes, known as hereditary cancers. These genes typically include tumor suppressors, DNA repair and replication fidelity genes, and occasionally oncogenes. In most hereditary cancer syndromes, Knudson’s two-hit hypothesis applies, where a second somatic event inactivates the remaining allele of a tumor suppressor or DNA repair gene, leading to tumorigenesis. Advancements in genome-wide sequencing have significantly enhanced our understanding of the mutational processes involved in hereditary cancers. In particular, the assessment of microsatellite instability (MSI), tumor mutational burden (TMB), and mutational signatures has emerged as a powerful tool for the identification of hereditary tumors. Tumors with high or ultra-high TMB often reflect underlying DNA repair deficiencies, while specific mutational signatures can pinpoint the defective pathway. These tumor mutational features are especially informative in syndromes involving mismatch repair (MMR), homologous recombination (HR), base excision repair (BER), nucleotide excision repair (NER), and polymerase proofreading. Moreover, tumor sequencing aids in the interpretation of germline variants, identifies somatic mosaicism, and helps differentiate hereditary from sporadic cancers. Additionally, tumor molecular features associated with DNA repair deficiencies offer insights into personalized therapies, such as the use of PARP inhibitors for *BRCA1/2*-deficient tumors and immune checkpoint inhibitors for MMR- and polymerase proofreading-deficient cancers. Tumor profiling also uncovers actionable mutations in oncogenes like *RET* and *VHL*, which can be targeted with specific therapies. This review explores the integration of tumor molecular features with germline genetic data to refine diagnosis, risk assessment, and therapeutic strategies in hereditary cancer.

## Introduction

Hereditary cancer syndromes account for a substantial subset of malignancies and are caused by germline pathogenic variants in genes that regulate cell proliferation, genomic stability, and DNA repair. Identifying individuals with an inherited cancer predisposition is critical, as it informs cancer risk assessment, surveillance strategies, treatment selection, and cascade testing of at-risk relatives. Traditionally, the diagnosis of hereditary cancer has relied primarily on germline genetic testing guided by personal and family history, tumor histopathology, and, for selected tumor types, limited molecular assays [1].

Advances in next-generation sequencing have fundamentally transformed this landscape by enabling tumor genomic profiling alongside germline analysis [2]. Tumors arising in the context of inherited cancer predisposition often harbor characteristic molecular features, such as somatic second-hit events or distinct mutational patterns associated with defects in DNA repair or replication fidelity pathways [3]. These tumor-derived features can provide important evidence for the presence of an underlying germline pathogenic variant, support variant interpretation, reveal somatic mosaicism, and help distinguish hereditary cancers from sporadic or non-genetic disease processes. Moreover, tumor molecular profiles increasingly inform therapeutic decision-making, particularly with the advent of targeted therapies and immunotherapy [4].

In this review, we examine how the integration of tumor molecular features with germline genetic data enhances the diagnosis and clinical management of hereditary cancer.

## Key players in hereditary cancer: tumor suppressor genes, DNA repair genes and oncogenes

**Fig. 1:**
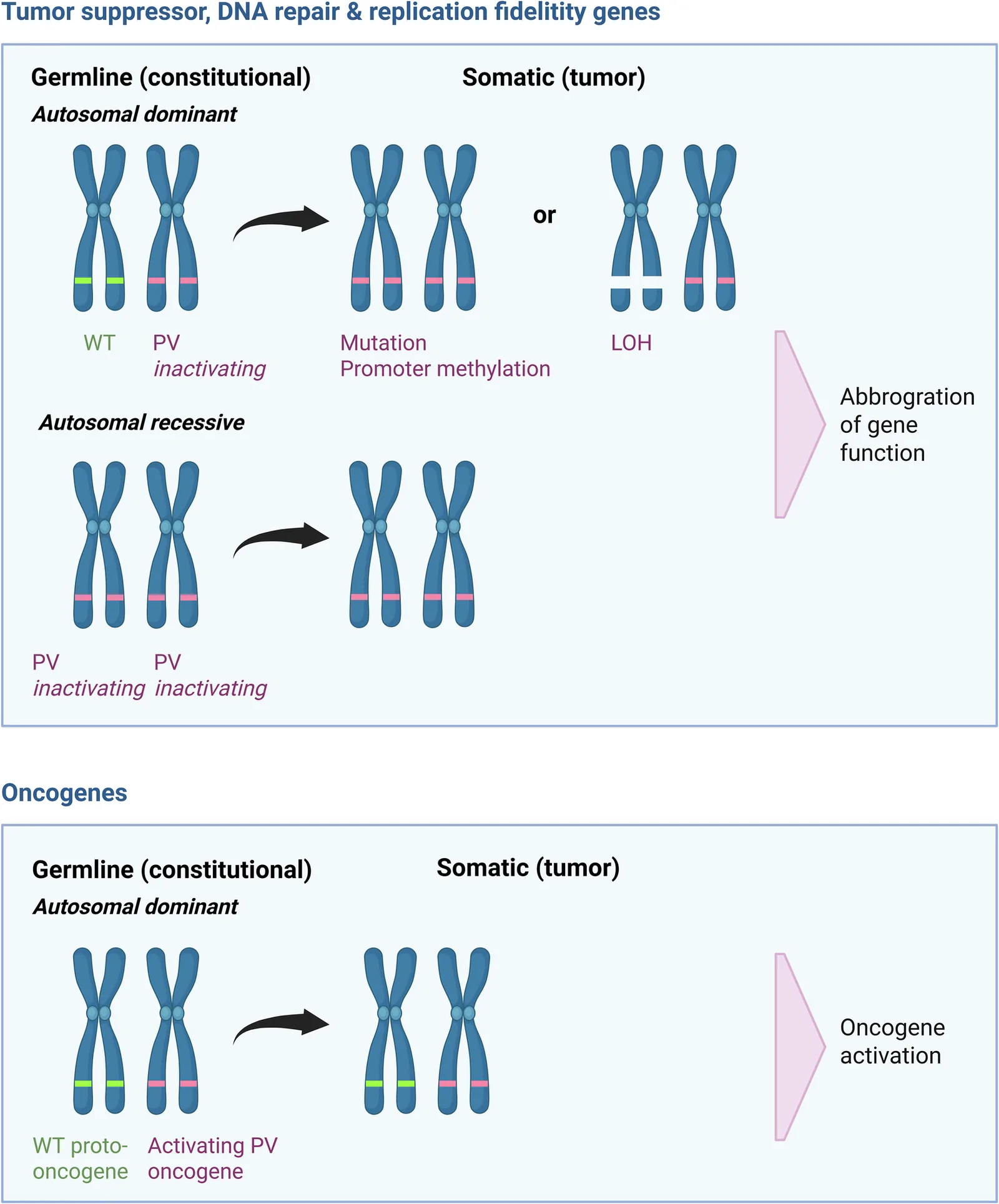
Genetic basis of hereditary cancer syndromes. Top panel: Mechanisms of inactivation of tumor suppressor, DNA repair and replication fidelity genes in hereditary cancer. In autosomal dominant syndromes, individuals inherit one pathogenic variant (PV), and somatic inactivation of the second allele (via mutation, promoter methylation, or LOH) leads to complete loss of gene function in the tumor, consistent with Knudson’s two-hit hypothesis. In autosomal recessive syndromes, biallelic inactivating variants are inherited. Bottom panel: Activation of oncogenes in hereditary cancer. A germline activating variant in a proto-oncogene can lead to oncogene activation. Created in https://BioRender.com. Abbreviations: *LOH* loss of heterozygosity, *PV* pathogenic variant, *WT* wildtype.

Hereditary cancers arise from germline pathogenic variants that confer an increased lifetime risk of tumor development. Known cancer predisposition genes fall into three major functional categories: tumor suppressor genes, DNA repair genes and oncogenes. Each class plays a distinct role in cellular homeostasis, and its disruption contributes to oncogenesis through different molecular mechanisms and inheritance patterns (Fig. 1).

### Tumor suppressor genes

The majority of hereditary cancer syndromes are caused by germline pathogenic variants in tumor suppressor genes, which normally encode proteins that inhibit cell proliferation, promote apoptosis, or maintain genomic stability. These genes act as cellular “brakes” to prevent uncontrolled growth [5].

In the context of hereditary cancer, individuals typically inherit or acquire (if de novo) one germline deleterious variant in one allele of a tumor suppressor gene. Following Knudson’s two-hit hypothesis [6], tumor development requires a second hit that inactivates the remaining functional allele, which often occurs through somatic mutation in the target tissue. A study of hereditary and sporadic retinoblastoma cases established the basis for the two-hit model: hereditary retinoblastoma arises from a germline pathogenic *RB1* variant followed by somatic inactivation of the second allele, whereas sporadic retinoblastoma results from two independent somatic hits in the gene [6]. This recessive behavior at the cellular level (despite autosomal dominant inheritance at the organism level) also underlies many well-characterized syndromes, such as those caused by germline pathogenic variants in *TP53*, *APC*, *NF1, VHL, STK11* or *PTEN*, among others [7].

### DNA repair and replication fidelity genes: a special class of tumor suppressors

A crucial subset of tumor suppressors includes DNA repair genes, which do not directly inhibit proliferation but instead maintain genomic integrity by correcting DNA damage [8]. Germline pathogenic variants in DNA repair genes (e.g., *MLH1*, *MSH2*, *ATM*, *BRCA1/2*, *MUTYH, NTHL1, POLE* or *POLD1*, among others) impair cells’ ability to correct replication errors or repair DNA breaks, leading to the gradual accumulation of mutations across the genome—including in key oncogenes and tumor suppressors, and/or causing genome instability, a recognized hallmark of cancer [9].

**Fig. 2:**
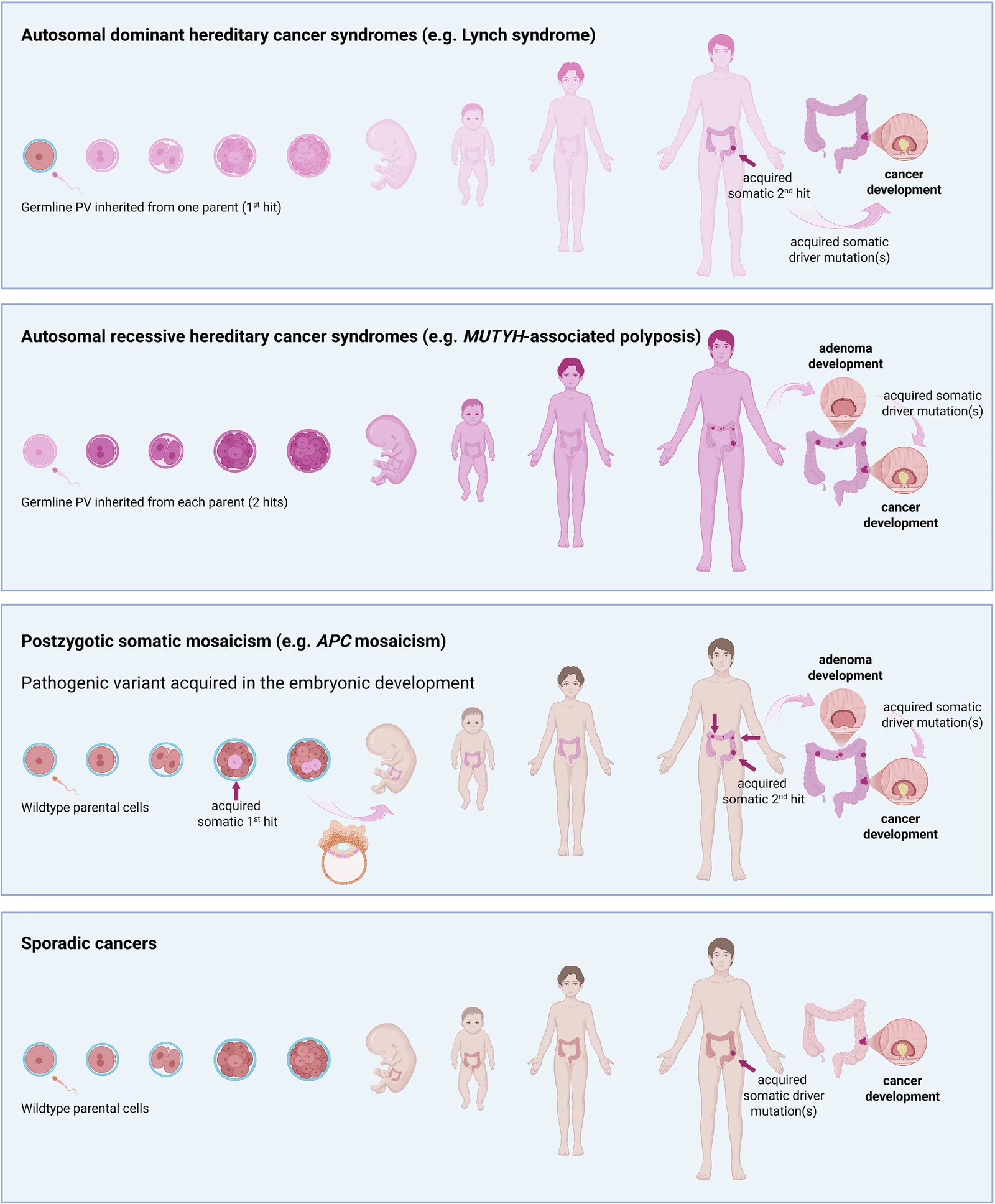
Genetic mechanisms underlying hereditary, mosaic, and sporadic cancer development. Schematic representation of the timing and origin of pathogenic variants leading to cancer. Top panel: Autosomal dominant hereditary cancer syndromes (e.g. Lynch syndrome), where a germline pathogenic variant (PV) constitutes the first hit and tumor development requires acquisition of a somatic second hit and additional driver mutations. Second panel: Autosomal recessive syndromes (e.g., *MUTYH*-associated polyposis), caused by biallelic germline pathogenic variants, with tumorigenesis following accumulation of somatic driver mutations. Third panel: Postzygotic somatic mosaicism (e.g., *APC* mosaicism), in which a pathogenic variant arises during embryogenesis, leading to mosaic tissues that may develop cancer after a somatic second hit. Bottom panel: Sporadic cancers, which arise exclusively through somatic driver mutations in the absence of germline pathogenic variants. Purple tonalities denote PV status, with lighter shades indicating monoallelic alteration, and darker shades representing biallelic alteration and/or additional somatic hits. Created in https://BioRender.com.

For most DNA repair genes, Knudson’s two-hit hypothesis applies: tumor development requires a second “hit” that results in the inactivation of both alleles (Table 1). In autosomal dominant syndromes, inactivation of the second allele typically occurs in the target tissues (Fig. 2). Examples include Lynch syndrome (caused by heterozygous pathogenic variants in *MLH1*, *MSH2*, *MSH6* or *PMS2*) and hereditary breast and ovarian cancer (*BRCA1* or *BRCA2*).

**Table 1:** Key features of variants and mechanisms that may affect tumor suppressor genes.

Feature	Two-hit hypothesis	Dominant Negative	Haploinsufficiency
**Alleles affected**	Both alleles (biallelic inactivation)	One allele (mutant interferes with the wild-type)	One allele (monoallelic inactivation)
**Mechanism**	Sequential inactivation (mutation, deletion, LOH, promoter methylation)	Active interference, mutant protein forms non-functional complexes with the wild-type protein	Reduced dosage
**Protein reduction**	Complete loss of function	Dysfunctional protein that blocks normal function	Less functional protein
**Common in**	Classical tumor suppressor genes and DNA repair genes	Multimeric proteins (e.g., transcription factors, enzymes)	Dosage-sensitive tumor suppressor genes
**Examples**	*TP53, APC, RB1,* MMR genes	*TP53, DNMT3A, RUNX1*	*PTEN, NF1, STK11, POLE*

*LOH* loss of heterozygosity.

In autosomal recessive cancer predisposition syndromes, both alleles are already inactivated in the germline (Fig. 2), such as in constitutional mismatch repair deficiency (CMMRD), caused by biallelic pathogenic variants in DNA mismatch repair genes (*MLH1*, *MSH2*, *MSH6*, or *PMS2*), *MUTYH*-, *NTHL1*-, or *MBD4*-associated adenomatous polyposes, Ataxia Telangiectasia (*ATM*), Fanconi Anemia (*FANCA*, *FANCC*, *FANCD2*), and Xeroderma Pigmentosum (*XPA*, *XPB*, *XPC*, *XPD*), among other rare recessive cancer syndromes [10, 11].

### Oncogenes

Although much less common in hereditary cancer, oncogenes—mutated or aberrantly activated forms of proto-oncogenes—also play a role. These genes encode proteins that promote cell proliferation and survival, such as growth factors, receptor tyrosine kinases, or signal transduction molecules [5]. Unlike tumor suppressor genes, oncogenes typically act in a dominant fashion at both the cell and organism levels. A single activating germline pathogenic variant is sufficient to predispose to cancer, as seen in rare syndromes like Multiple Endocrine Neoplasia type 2 (*MEN2*), caused by gain-of-function variants in the *RET* proto-oncogene [12], or in gastrointestinal stromal tumors (GISTs), where gain-of-function variants in the *KIT* proto-oncogene lead to tumorigenesis [13].

## Knudson’s two-hit hypothesis revisited

As aforementioned, most tumor suppressor genes follow Knudson’s two-hit hypothesis, meaning that two “hits” (e.g., events that inactivate a single allele) are required to inactivate both alleles and initiate tumor formation [6]. The presence of a germline pathogenic variant (or two, in the case of recessive disorders) as the first hit is the underlying reason why cancers in the hereditary context tend to develop earlier in life than sporadic (i.e., non-hereditary) cancers (Fig. 2). It also explains why multiple primary tumors may develop in a single individual (e.g., colon and endometrial cancer, or multiple primary colorectal tumors in Lynch syndrome), or why bilateral tumors can occur in paired organs (e.g., both breasts in hereditary breast and ovarian cancer, or both kidneys in Von-Hippel Lindau syndrome) [14].

While Knudson’s two-hit model is fundamentally correct for initiating tumorigenesis, over the past decades, we have learned that the process is far more complex. The inactivation of a tumor suppressor gene is a crucial step, but the progression from a benign tumor to an invasive carcinoma with the potential to metastasize requires additional genomic changes. This progression has been well-described for many cancers, such as colorectal cancer, where inactivation of *APC* typically occurs first, followed by alterations in other tumor suppressor genes (e.g., *TP53*) and oncogenes (e.g., *KRAS*), each contributing to further progression into a colorectal carcinoma [15, 16].

A characteristic feature of inherited cancer predisposition syndromes is the pronounced tissue specificity of cancer risk, whereby germline pathogenic variants confer high susceptibility to malignancy in one or a limited number of tissues while sparing others. This phenomenon reflects the interplay between the inherited genetic defect and tissue-dependent biological contexts. Contributing factors include tissue-specific gene expression and function, differences in stem cell dynamics and proliferative rates, exposure to distinct endogenous or environmental mutational processes, and the requirement for particular cooperating somatic events that arise preferentially in certain cell types [17–19]. In addition, epigenetic and chromatin landscapes, local microenvironmental contexts, and immune surveillance mechanisms further modulate the oncogenic consequences of germline variants in a tissue-restricted manner. Together, these factors help explain why broadly expressed cancer susceptibility genes can drive malignancy selectively in specific organs, a question that remains an active area of investigation.

### Modern perspectives: dominant-negative effects and haploinsufficiency

Knudson’s two-hit hypothesis does not universally apply to all tumor suppressor genes, as some genes do not always require two independent hits to interfere with their function. Occasionally, tumor suppressors may be inactivated by the presence of dominant negative variants. These variants produce proteins that not only lose normal function but also interfere with the function of the wild-type protein transcribed from the other allele. Thus, a single allele mutation can disrupt the entire protein complex. While *TP53* is traditionally inactivated by the two-hit mechanism, it is also a well-known example of a protein subject to dominant-negative variants in cancer. As a tetrameric transcription factor, a dominant-negative missense mutation in TP53’s DNA-binding domain can prevent the tetramer from binding DNA or activating target genes (e.g., *CDKN1A* and *BAX*), resulting in complete or significant cellular dysregulation [20, 21]. This dysregulation can confer a significant growth advantage or increased survival capabilities to a cell, which may explain why many cancers harbor missense mutations in *TP53* rather than simple inactivating alterations. Additionally, dominant-negative mutations in *TP53* may contribute to therapy resistance, as mutant *TP53* can actively suppress stress response pathways [22]. Other examples of dominant-negative variants in cancer include *DNMT3A* (DNA methyltransferase 3 A) and *RUNX1* (hematopoietic transcription factor) in specific forms of leukemia [23, 24].

Another form of tumor suppressor gene inactivation is haploinsufficiency, where the loss of one functional allele is insufficient to maintain normal protein function, contributing to tumor development. Unlike dominant-negative mutations, the mutant allele does not interfere with the wildtype protein; rather, the reduced dosage of the protein is inadequate for its tumor-suppressive role (Table 1). Mechanistically, these genes are dosage-sensitive, requiring the expression of both alleles to produce sufficient protein for normal cellular function. A reduced gene dosage can impair critical processes such as DNA repair, cell cycle control, and apoptosis, thereby promoting tumorigenesis. Examples of genes where haploinsufficiency plays a role include *PTEN*, *STK11*, *NF1* and *POLE* [25–28].

### DNA repair deficiencies and their tumor mutational features

In addition to somatic mutations that contribute directly to cancer development, i.e., driver mutations that inactivate tumor suppressor genes or activate oncogenes, tumors can accumulate many other mutations. These genetic variants may arise from exposure to mutagens like tobacco smoke, ultraviolet radiation, and aflatoxin, or from intrinsic mutagenic processes, such as DNA replication errors, spontaneous deamination, and defective DNA repair [3, 29].

The total number of mutations in a cancer genome is often quantified as the tumor mutational burden (TMB), typically expressed as the number of somatic mutations per megabase (mut/Mb) [30]. TMB can be calculated across the entire genome or limited to non-synonymous mutations in coding regions. TMB can also serve as a proxy for genomic instability at the base pair level. High TMBs suggest either DNA repair deficiencies or the involvement of specific mutagenic processes (extrinsic or intrinsic) during cancer development. Interestingly, TMB has gained clinical relevance as a potential biomarker for predicting responses to certain therapies [30]. which is discussed further in Section “Tumor features as biomarkers and therapeutic implications”.

Beyond assessing the mutational burden, the specific types of mutations within the cancer genome can provide insights into the biological processes that shaped the cancer’s evolution. Mutations arising from a particular mutagenic process can be identified as mutational signatures, which are defined by distinct patterns of base substitutions, insertions, deletions, and structural variants and their sequence context [3, 31]. Nowadays, mutational signatures can be computationally deconvoluted, helping to identify the underlying mutagenic processes. To date, more than a hundred distinct mutational signatures have been described, and for approximately half of these, the associated mutagenic process has been identified (https://cancer.sanger.ac.uk/signatures/; https://analysistools.cancer.gov/mutational-signatures/#/; https://signal.mutationalsignatures.com/explore/main/cancer/signatures).

### DNA repair deficiencies in hereditary cancer

In the early 1990s, deficiencies in the DNA mismatch repair (MMR) genes *MSH2* and *MLH1* were the first identified genetic defects in DNA repair genes that were linked to hereditary cancer. The associated syndrome—originally termed *hereditary non-polyposis colorectal cancer* (HNPCC) and currently known as *Lynch syndrome*—had already been clinically described in the 1960s by Dr. Henry Lynch [32]. Subsequent sequencing of germline and tumor DNA revealed pathogenic germline variants and somatic alterations, respectively, in *MSH2* and *MLH1*, and later in *MSH6* and *PMS2*. These genes are inactivated following Knudson’s two-hit hypothesis, establishing defective DNA repair as a key mechanism in hereditary cancer. Sequencing of tumors from patients with Lynch syndrome, as well as from sporadic colorectal cancer cases, identified microsatellite instability (MSI) as a hallmark molecular feature, characterized by alterations in short, repetitive DNA sequences due to MMR deficiency. By the late 1990s, standardized assays for MSI testing were developed, and it was soon demonstrated that MSI status served as a prognostic marker in early-stage CRC [32]. The discovery of MMR deficiency and MSI laid the conceptual foundation for the TMB. However, TMB could not be accurately quantified across cancer genomes until the late 2000s and early 2010s, with the advent of next-generation sequencing (NGS). Tumors exhibiting MSI typically harbor high TMB, with mutational loads ranging from approximately 10 to 200 mut/Mb [33] (Table 2).

**Table 2:** Mutational signatures associated with defective DNA repair and replication fidelity in genetic tumor risk syndromes and their associated tumor types.

DNA repair or replication fidelity mechanism	Genes involved	Associated genetic tumor risk syndrome	TMB	Mutational signatures	Major tumor types	References
Mismatch repair (MMR)	*MLH1, MSH2, MSH6, PMS2*	Lynch syndrome (autosomal dominant)	High	SBS6, SBS14*, SBS15, SBS20**, SBS21, SBS26 (*PMS2*), SBS44, SBS97, DBS7, DBS10, DBS14*,DBA29, DBS37, ID7, ID16a*, ID16b*, ID19, ID20*, ID21**	Colorectal, endometrial	Alexandrov et al. 2020 [3]; Degasperi et al. 2022 [72]; Koh et al. 2025 [42]
		Constitutional mismatch repair deficiency (CMMRD; autosomal recessive)	Ultra-high	SBS14*, SBS15, SBS20, SBS26, ID1, ID2, ID12, IDA^#^	Colorectal, brain, hematological	Ercan et al. 2024 [73]; Weijers et al. 2025 [74]
Homologous recombination (HR)	*BRCA1, BRCA2, PALB2*	Hereditary breast and ovarian cancer (HBOC; autosomal dominant)	Moderate	SBS3, ID6, high structural variant burden	Breast, ovarian	Nik-Zainal et al. 2016 [75] and 2021 [76]; Davies et al. 2017 [77]
	*FANCA*, *FANCB, FANCC, FANCD1, FANCE, FANCF, XRCC9* and other genes involved in homologous recombination	Fanconi anemia (FA; autosomal recessive)	Moderate	SBS1, SBS2, SBS5, SBS8, SBS13, SBS18, high structural variant burden	Head and neck, esophageal and anogenital squamous cell carcinomas	Webster et al. 2022 [78]
Base excision repair (BER)	*MUTYH, NTHL1, MBD4*	*MUTYH*-associated polyposis, *NTHL1*-associated tumor syndrome, *MBD4*-associated neoplasia syndrome	Moderate	SBS36 (*MUTYH*), SBS30 (*NTHL1*), SBS96 (*MBD4*)	Colorectal (all), breast (*NTHL1*), acute myeloid leukemia (*MBD4*)	Pilati et al.2017 [79]; Silveira et al. 2024 [80]; Grolleman et al. 2019 [81]; Palles et al. 2024 [37]; Degasperi et al. 2022 [72]
Nucleotide excision repair (NER)	*DDB2, ERCC1, ERCC2, ERCC3, ERCC4, ERCC5, POLH, XPA, XPC*.	Xeroderma Pigmentosum (XP; autosomal recessive)	Moderate	SBS8	Skin	Jager et al. 2019 [82]
Polymerase proofreading	*POLE, POLD1*	Polymerase proofreading-associated polyposis	Ultra-high	*POLE*: SBS10a, SBS10b, SBS28, SBS14*, DBS3, DBS10, DBS14*, ID15, ID16a*, ID16b*, ID20* *POLD1*: SBS10c, SBS10d, SBS20**, ID14, ID21**	Colorectal, endometrial	Degasperi et al. 2022 [72]; Mur et al. 2023 [39]; Koh et al. 2025 [42]

^*^Mutational signatures observed in tumors that are defective of both polymerase epsilon (*POLE*) and DNA mismatch repair.

**Mutational signatures are observed in tumors that are defective of both polymerase delta (*POLD1*) and DNA mismatch repair.

^#^No mutational catalogue signature assigned [74].

In addition to MMR, several other DNA repair and replication fidelity pathways have been implicated in hereditary cancer syndromes over the past three decades. The homologous recombination (HR) pathway, involving key genes such as *BRCA1*, *BRCA2*, and *PALB2*, plays a central role in maintaining genomic stability through the repair of DNA double-strand breaks. Monoallelic germline pathogenic variants in these genes confer susceptibility to hereditary breast and ovarian cancer, with additional risks for pancreatic and prostate cancers depending on the specific gene involved [34]. The base excision repair (BER) pathway includes genes such as *MUTYH*, *NTHL1*, and *MBD4*, in which biallelic germline inactivation leads to distinct colorectal polyposis syndromes, each associated with variable extracolonic cancer risks depending on the specific gene involved [35–37]. The nucleotide excision repair (NER) pathway includes genes such as *XPA*, *XPC*, and *ERCC2*, which are essential for the removal of bulky DNA adducts and helix-distorting lesions, such as those caused by ultraviolet (UV) radiation. Biallelic germline pathogenic variants in these genes cause syndromes such as Xeroderma Pigmentosum (XP), characterized by extreme sensitivity to UV radiation and a significantly elevated risk of skin cancers [38]. Lastly, the polymerase proofreading mechanism is governed by the exonuclease activity of DNA polymerases ε and δ (*POLE* and *POLD1* genes). These enzymes not only synthesize new DNA strands but also correct replication errors by removing and correcting misincorporated nucleotides. Monoallelic germline pathogenic variants in the exonuclease domains of these polymerases result in polymerase proofreading-associated polyposis (PPAP), characterized by multiple colorectal adenomas and increased risks of colorectal and endometrial cancers [28, 39].

Genome-wide assessment of the somatic mutational landscape of tumors with deficiencies in these DNA repair and replication fidelity pathways has revealed a spectrum of TMB. Tumors associated with BER and HR deficiencies typically exhibit moderate TMB, those with MMR and NER deficiencies show high TMB, and cancers with polymerase proofreading defects usually display ultra-high TMB (Table 2).

### Mutational signatures in hereditary cancer

The mutagenic consequences of defective DNA repair and replication fidelity in cancer can be more precisely identified through the assessment of mutational signatures, in addition to TMB. To date, the mutational signature landscape for the major genes involved in DNA repair and replication fidelity has been extensively studied [3, 40]. Until approximately five years ago, this analysis was primarily focused on single base substitution (SBS) signatures, in which substitutions occur within a specific sequence context (e.g., the flanking bases) that determines the resulting mutational pattern. More recently, the analysis has expanded to include doublet base substitution (DBS) signatures, which involve substitutions of two adjacent bases; insertion and deletion (ID) signatures, which represent small insertions or deletions of nucleotides; and copy number (CN) and structural variant (SV) signatures, which reflect changes in the copy number of DNA segments or may indicate genomic rearrangements such as inversions, duplications, and translocations [41, 42]. Through technologies such as exome sequencing, short-read genome sequencing, and, ideally, long-read genome sequencing, a vast catalog of mutational signatures associated with hereditary cancer has been compiled. A summary of these signatures linked to defective DNA repair and replication fidelity in genetic tumor risk syndromes is provided in Table 2.

Analyzing mutational signatures can be a powerful tool for the identification of genetic tumor risk syndromes, particularly when traditional genetic testing is inconclusive or when multiple tumors are present. The detection of specific mutational signatures, such as those described in Table 2, can provide immediate insights into which DNA repair or replication fidelity pathway is defective and suggest the likely affected gene harboring germline pathogenic variants. Furthermore, mutational signature analysis can aid in interpreting germline variants of uncertain significance (VUS), such as novel variants in the exonuclease domain of *POLE or POLD1*. The presence of mutational signatures associated with polymerase ε (*POLE)* or δ *(POLD1)* proofreading deficiency in a tumor may provide functional evidence for exonuclease defects and support the pathogenicity of the identified variant in the corresponding gene [39].

## Leveraging tumor sequencing to refine germline variant interpretation

Tumor sequencing results can aid in the classification of variants when incorporated into gene-specific variant interpretation frameworks. Tumor-based information may be incorporated into ACMG/AMP classification criteria, specifically criteria PP4 (supporting pathogenicity) and BP5 (supporting benignity), which consider the patient’s phenotype, including tumor molecular features. The ClinGen Variant Curation Expert Panels (VCEPs) for DNA repair and replication fidelity genes are formalizing recommendations for the use of tumor molecular data in variant classification. Currently, validated tumor features are incorporated into variant classification recommendations for MMR genes (*MLH1, MSH2, MSH6*, and *PMS2*). These include MMR deficiency identified through PCR- or NGS-based MSI testing or immunohistochemistry (IHC), as well as the presence of *BRAF* V600E mutations or *MLH1* promoter hypermethylation—both of which are considered evidence supporting benign classification of germline *MLH1* variants (https://cspec.genome.network/cspec/ui/svi/affiliation/50099). In addition, draft recommendations from the corresponding ClinGen VCEP are under development and will incorporate tumor mutational signature results for the interpretation of variants in *POLE* and *POLD1* (exonuclease domain) and *NTHL1*. Outside the ClinGen framework, existing guidelines for *POLE* and *POLD1* variant classification already support the use of tumor mutational signatures [39].

## When tumor data helps identify non-inherited conditions

### Post-zygotic somatic mosaicism

Post-zygotic somatic mosaicism occurs when mutations arise de novo in a subset of cells after fertilization, either during early embryonic development or later in life. This results in the presence of genetically distinct cell populations within the same individual [43] (Fig. 2). Mosaic variants in hereditary cancer genes can be easily missed during routine germline testing, particularly when the pathogenic variant is absent in hematopoietic cells or present at very low allele fractions. Sequencing of the tumor(s) and/or normal target tissues may help detect previously missed mosaic variants, confirm mosaicism when a pathogenic variant was detected with low allele fraction in blood-derived DNA, or distinguish mosaicism from clonal hematopoiesis or circulating tumor cells or DNA [2, 44].

Testing for the mosaic variant using highly sensitive, locus-specific detection methods -such as digital PCR- on DNA from multiple sources/lineages can help confirm the diagnosis and distinguish between localized mosaicism (restricted to one or a few organs) and soma-wide mosaicism. This distinction has important implications for cancer risk assessment and the development of personalized surveillance and management strategies. Post-zygotic somatic mosaicism can occur in any hereditary cancer gene; however, the most frequently reported genes in which mosaicisms have been detected include *APC*, *NF1*, *NF2*, *RB1*, *TP53*, and *VHL*. Nevertheless, mosaicisms affecting common hereditary cancer genes (e.g., Lynch syndrome genes or *BRCA1/2*) are also identified [44, 45].

Generally, individuals with somatic mosaicism do not have a family history of the associated disease (in previous generations or among siblings). However, a recent study reported an exception in which two first-degree relatives in different generations—a father and his daughter—were affected by adenomatous polyposis, each exhibiting somatic mosaicism involving distinct, non-shared *APC* variants [46]. This observation appears to be coincidental (and likely extremely exceptional) rather than indicative of a shared inherited genetic cause.

### Aberrant clonal expansions that may confound germline testing interpretation: clonal hematopoiesis and circulating tumor cells or DNA

Clonal hematopoiesis (CH) arises when somatic mutations in hematopoietic stem cells provide a fitness advantage and lead to clonal expansion. Individuals with expanded mutated clones in blood cells are at higher risk of hematological cancer and detrimental cardiovascular and pulmonary health through proinflammatory conditions. Aging, cancer therapy and smoking are among the factors associated with the presence of CH [47].

While most cases of CH involve a limited number of genes, most commonly *DNMT3A*, *TET2* and *ASXL1*, some hereditary cancer genes are also recurrent targets of CH, particularly *TP53*, *ATM* and *CHEK2* (refs. 47–49). Routine germline testing for hereditary cancer syndromes may incidentally detect CH-associated pathogenic variants in these or other genes, potentially leading to misclassification as germline pathogenic variants or as somatic mosaicism—particularly when the variant is detected at a reduced allele fraction. In theory, a similar scenario may occur in the presence of circulating tumor cells or tumor-derived cell-free DNA in the blood, which can also confound germline test interpretation. However, likely due to the expected extremely low allele fraction, this has not yet been described in the literature.

In these contexts, sequencing of tumor tissue and/or DNA derived from non-hematopoietic lineages (buccal epithelial cells, hair follicles, nails, skin fibroblasts, or any mesoderm- or ectoderm-derived tissue) is critical for accurately discriminating pathogenic variants attributable to CH (or circulating tumor-derived DNA) from bona fide germline pathogenic variants or post-zygotic somatic mosaicism.

### Somatic (tumor)-only events

As discussed in previous sections, the identification of tumor mutational signatures or molecular features associated with specific DNA repair deficiencies can raise suspicion for an underlying hereditary cancer syndrome. However, these tumor features may also result from somatic mutations or epigenetic alterations (promoter hypermethylation) in the corresponding DNA repair genes—changes that are not associated with germline pathogenic variants. This scenario is observed across all DNA repair genes implicated in hereditary cancer syndromes, where somatic mutations or epimutations are also found in sporadic cancers (e.g., *POLE*, *BRCA1/2*, MMR genes, or *MLH1* promoter hypermethylation).

A clear example of the clinical utility of tumor sequencing is in the evaluation of MMR-deficient tumors without *MLH1* promoter hypermethylation. In such cases—particularly in colorectal cancer—the absence of somatic *MLH1* promoter methylation typically raises strong suspicion for Lynch syndrome. However, germline testing may sometimes fail to identify pathogenic variants, leaving the possibility open of an undetected germline alteration (e.g., deep intronic, regulatory, or structural variants not detected by standard testing) [50]. In these cases, tumor sequencing can provide critical clarification: if biallelic somatic inactivation of an MMR gene is identified (via mutations and/or LOH), it supports a sporadic origin of the MMR deficiency rather than an undetected germline pathogenic variant, effectively ruling out Lynch syndrome. Of note, although highly unlikely, the possibility of a phenocopy cannot be entirely excluded.

### Non-genetic etiologies that leave a mutational footprint

Certain treatments—particularly chemotherapy and radiation—can induce mutations in the DNA of both healthy and malignant cells. While most cells sustaining treatment-related DNA damage undergo cell death, some may survive, potentially giving rise to secondary malignancies [51–53]. This risk is especially relevant in pediatric and young-onset cancer survivors, as well as in individuals exposed to recurrent ionizing radiation (e.g., multiple diagnostic X-rays) [53]. Platinum-based agents, capecitabine/5-fluorouracil (5-FU), alkylating agents (e.g., temozolomide), and radiation therapy have all been associated with distinct tumor mutational signatures [3, 54, 55]. The occurrence of multiple primary tumors is a key criterion for suspecting a hereditary cancer predisposition. However, when therapy-related mutational signatures are identified in metachronous primary tumors diagnosed years after a previous malignancy—particularly when the first cancer was treated with radiation or the aforementioned chemotherapies—this pattern supports a therapy-associated etiology rather than an underlying genetic predisposition.

Nevertheless, these findings should be interpreted with caution, as certain oncologic treatments (radiotherapy or specific chemotherapeutic agents) can increase the risk of secondary neoplasms in patients with hereditary syndromes associated with DNA repair deficiency. These metachronous tumors may also exhibit therapy-related mutational signatures. Such syndromes include the recessive disorders Fanconi Anemia (FA genes), Bloom syndrome (*BLM*), Ataxia-Telangiectasia (*ATM*), Nijmegen Breakage Syndrome (*NBN*), and Xeroderma Pigmentosum (XP genes), as well as Li-Fraumeni syndrome (*TP53*) and, to a lesser extent, hereditary breast and ovarian cancer (*BRCA1*, *BRCA2*).

A substantial proportion of colorectal cancers may be associated with alterations in the gut microbiome (dysbiosis). *Escherichia coli* is a common component of the normal intestinal microbiome; however, certain strains have acquired virulent factors that make them pathogenic. A subset of these pathogenic *E. coli* strains carries the *pks* pathogenicity island, which encodes the enzymatic machinery required for the synthesis of colibactin—a chemically unstable, non-ribosomal peptide–polyketide hybrid secondary metabolite with genotoxic properties. Colibactin induces DNA damage by causing interstrand cross-links and double-strand breaks, which may lead to mutations in tumor suppressor genes (e.g., *APC*) or oncogenes, leading to carcinogenesis [56]. *pks* + *E. coli* is now recognized as a driver of colorectal cancer, with approximately 9%-12% of colorectal cancers exhibiting colibactin-associated COSMIC mutational signatures SBS88 and ID18 (ref. [57, 58]). A priori, the identification of these mutational signatures contributing substantially to the mutational landscape of colorectal tumors supports a pks⁺ microbial etiology, rather than a hereditary cancer predisposition.

On the other hand, Dejea et al. reported that bacterial genes encoding colibactin (clbB) and *Bacteroides fragilis* toxin (bft) were enriched in the colonic mucosa of patients with Familial Adenomatous Polyposis (FAP), i.e., individuals harboring germline pathogenic variants in *APC*, compared with healthy controls. This enrichment was attributed to the presence of patchy bacterial biofilms containing these toxin-producing species [59]. In a more recent study, Stratton et al. performed whole-genome sequencing of 279 colonic crypts from FAP patients. They identified multiple mutational signatures in histologically normal crypts, including SBS88 and ID18, whose contribution was similar to that observed in normal crypts from non-carriers of germline *APC* pathogenic variants (healthy donors). Moreover, neither SBS88 nor ID18 was enriched in aberrant crypt foci from FAP patients [60]. Collectively, these findings suggest that bacterial genotoxins do not constitute the primary driver of carcinogenesis in this syndrome. Sequencing of FAP-associated colorectal cancers will determine whether colibactin-associated mutational signatures are present, and if so, to what extent.

In conclusion, although bacterial biofilms enriched in colibactin-producing *E. coli* have been identified more frequently in the colonic mucosa of FAP patients than in healthy controls, current genomic evidence does not support a primary etiological role for colibactin-driven mutagenesis in FAP-associated tumorigenesis. The evidence available to date suggests that colibactin-associated mutational signatures in colorectal cancers (SBS88 and ID18) predominantly arise in sporadic tumors, supporting a microbial rather than hereditary origin.

## Tumor features as biomarkers and therapeutic implications

Over the past few decades, significant insights have been gained into the DNA repair and replication fidelity pathways critical for the development of hereditary cancer. Interestingly, these insights have not only expanded our understanding of tumorigenesis but also opened up possibilities for therapeutic interventions. Cancers arising from deficiencies in *BRCA1*, *BRCA2*, and *PALB2*, and by extension defects in other HR genes, have been shown to be sensitive to platinum-based therapies and poly(ADP-ribose) polymerase (PARP) inhibitors (PARPi) [61, 62]. Recent studies examining the mutational landscape of tumors with HR gene deficiencies have revealed that, in addition to hereditary HR-deficient breast and ovarian cancer, other associated HR-deficient cancer types, including pancreatic, prostate, and colorectal cancers, may also benefit from these therapies [63, 64]. Moreover, the development of immune checkpoint inhibitors (ICI) has revolutionized the treatment of cancers with a high TMB, including not only sporadic cancers such as colorectal cancer, endometrial cancer, lung cancer and melanoma, among others, but also hereditary cancers associated with deficiencies in MMR, polymerase proofreading, and, potentially, BER [4, 65–67]. For example, cancers arising in the context of Lynch syndrome can benefit from ICI treatment, as can rarer forms of biallelic mismatch repair deficiency causing CMMRD [68, 69]. Additionally, case reports suggest that individuals with tumors resulting from *MBD4* deficiency may also benefit from ICI, with notable improvements in overall survival in patients with uveal melanoma [66].

Beyond therapies targeting the indirect effects of DNA repair and replication fidelity deficiencies, advances in genomic profiling have revealed actionable mutations in other hereditary cancer contexts. For instance, pathogenic variants in *RET*, implicated in MEN2, can be targeted with selective RET inhibitors [70], and individuals with germline *VHL* pathogenic variants associated with renal tumors respond to HIF-2α inhibitors [71]. By integrating germline genetics with tumor-specific features and leveraging advances in targeted therapies developed for sporadic cancers, personalized therapies can be offered to individuals with hereditary cancers. These therapies are more effective than conventional chemotherapy or radiation and exemplify the potential of precision medicine, where treatment is tailored not only to the cancer type but to the unique genetic makeup of each patient.

## Technical considerations

While combined germline and tumor genomic analyses hold great promise for cancer diagnostics and personalized treatment, germline and tumor sequencing differ substantially with respect to sample acquisition, preparation, variant interpretation, and clinical follow-up. Ideally, genomic DNA isolated from blood and from fresh/frozen tumor tissue is used. However, whereas the use of blood-derived genomic DNA is common practice, tumor profiling most often relies on sequencing DNA extracted from formalin-fixed paraffin-embedded (FFPE) tumor tissue. The use of FFPE-derived DNA introduces sequencing artifacts, primarily due to spontaneous cytosine deamination, which can distort somatic variant detection and subsequent mutational signature analyses. In addition, DNA in FFPE tissues often becomes highly degraded over time, limiting the utility of older specimens.

To mitigate these issues, optimized laboratory protocols—including enzymatic DNA repair and the use of unique molecular identifiers (UMIs)— as well as tailored bioinformatic approaches, such as using Strelka and MuTect for variant detection and FFPEsig for computational correction of artifacts in mutational signature analysis, can aid in the identification and filtering of sequencing artifacts.

## Conclusions and future directions

**Fig. 3:**
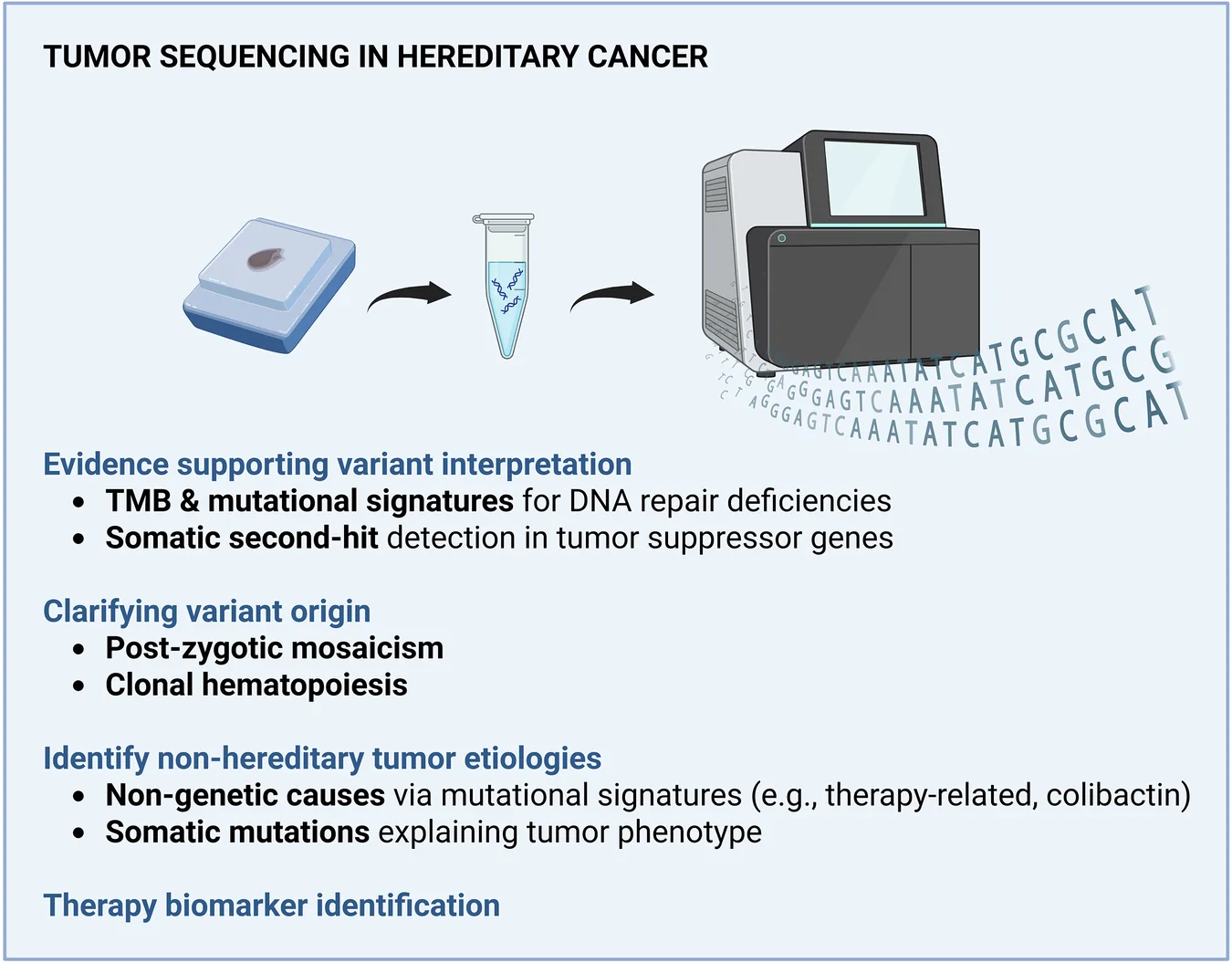
Uses of tumor sequencing in hereditary cancer. Integrated germline and tumor genomic sequencing supports germline variant interpretation, determination of variant origin (e.g., post-zygotic mosaicism, clonal hematopoiesis), clarification of tumor etiology, and identification of clinically relevant therapeutic biomarkers. TMB tumor mutational burden. Created in https://BioRender.com.

The convergence of germline and tumor genomic analyses has profoundly transformed our understanding of hereditary cancer. Tumor molecular profiling—including the assessment of MSI, TMB, and mutational signatures—now serves not only as a window into the mutational processes driving tumorigenesis, but also as a diagnostic, prognostic, and therapeutic tool. Integrating germline and somatic data enables the identification of pathogenic variants that might otherwise go undetected (e.g., those arising from post-zygotic mosaicism), the differentiation between true germline alterations and clonal hematopoiesis, the clarification of variants of uncertain significance, and the distinction between hereditary, sporadic, or non-genetic forms of disease (Fig. 3).

The study of mutational signatures has been particularly transformative, revealing the distinct molecular fingerprints of deficiencies in DNA repair and replication fidelity pathways such as MMR, HR, BER, NER, and polymerase proofreading. These signatures not only facilitate the identification of underlying genetic defects but also support variant interpretation frameworks and patient stratification for targeted therapies. As sequencing technologies and computational tools continue to evolve, the detection of complex mutational patterns—including doublet substitutions, indels, and structural variants—will become increasingly precise, enabling a more nuanced dissection of DNA repair defects and environmental or therapy-induced mutational processes.

Importantly, the integration of tumor molecular features into clinical practice has expanded the possibilities for personalized therapy. The use of PARP inhibitors in HR-deficient tumors and immune checkpoint inhibitors in MMR-, polymerase proofreading-, and potentially BER-deficient cancers exemplifies the translation of molecular insights into tailored treatments. Similarly, the identification of actionable germline variants in genes such as *RET* and *VHL* underscores the potential of precision oncology in hereditary cancer management.

Future efforts should focus on the systematic incorporation of tumor molecular data into germline variant interpretation frameworks, standardization of bioinformatic pipelines for mutational signature analysis in the clinical setting, and the study of diverse populations and tumor types. Expanding access to paired tumor–normal sequencing in clinical practice is critical for improving diagnostic accuracy, informing surveillance strategies, and guiding treatment selection. Finally, the incorporation of longitudinal genomic data, epigenetic profiling, and environmental exposure histories will provide a more comprehensive understanding of how inherited and acquired factors interact to influence cancer risk.
